# Supplementary figures and images for: Hydrogen sulfide blocks HIV rebound by maintaining mitochondrial bioenergetics and redox homeostasis
Source: eLife. 2021 Nov 18;10:e68487. doi: 10.7554/eLife.68487 (PMC8660018; doi:10.7554/eLife.68487)

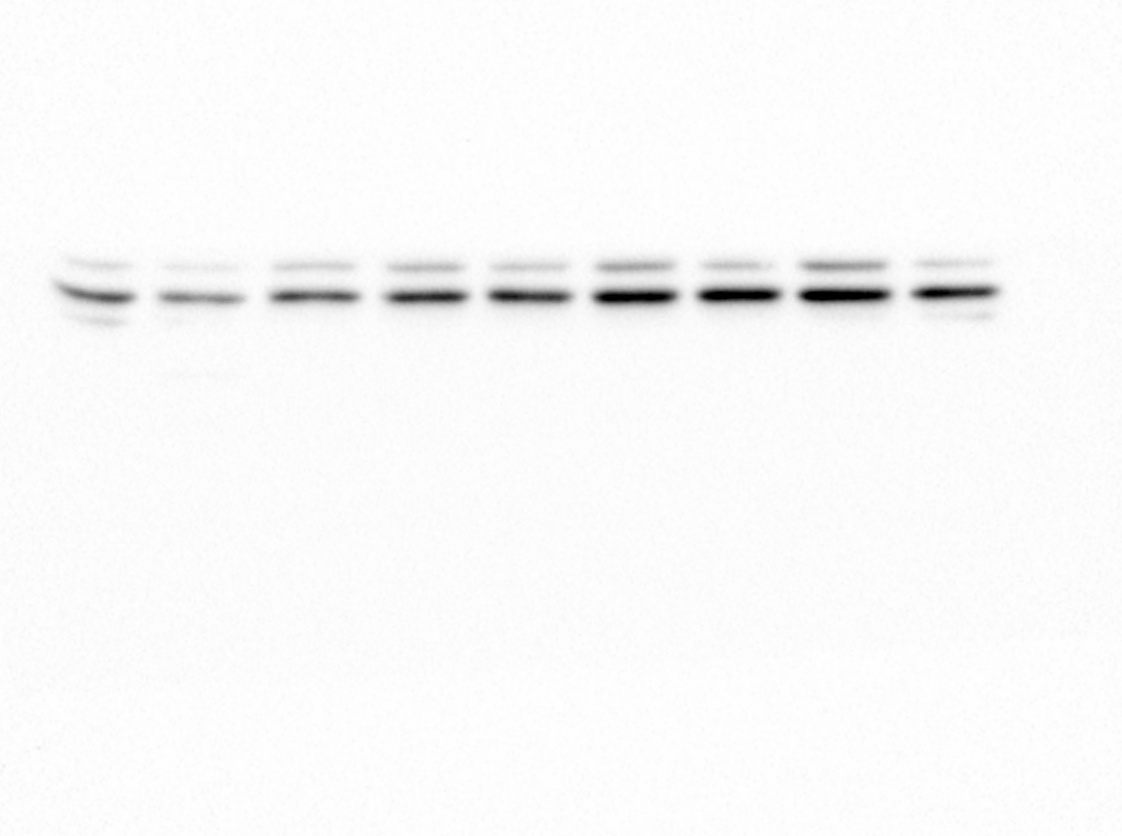

Supplement: Figure 1—source data 1. [file elife-68487-fig1-data1.zip › Figure 1-source data1/U1+PMA_MPST.jpg]

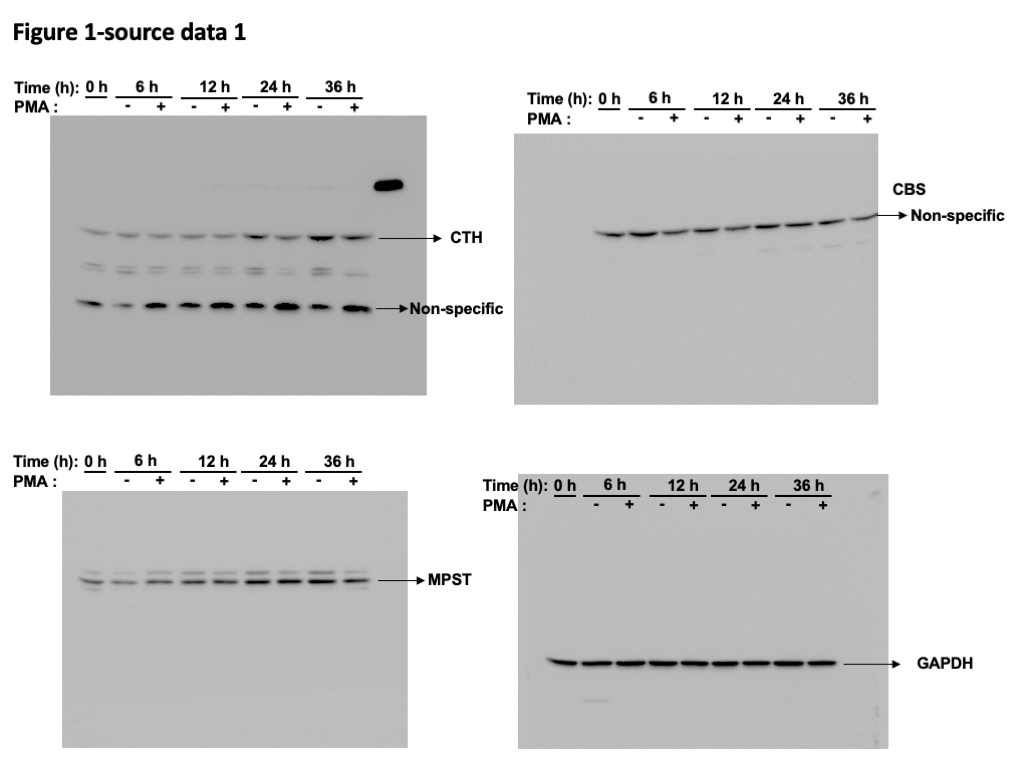

Supplement: Figure 1—source data 1. [file elife-68487-fig1-data1.zip › Figure 1-source data1/Figure 1-source data 1.tiff]

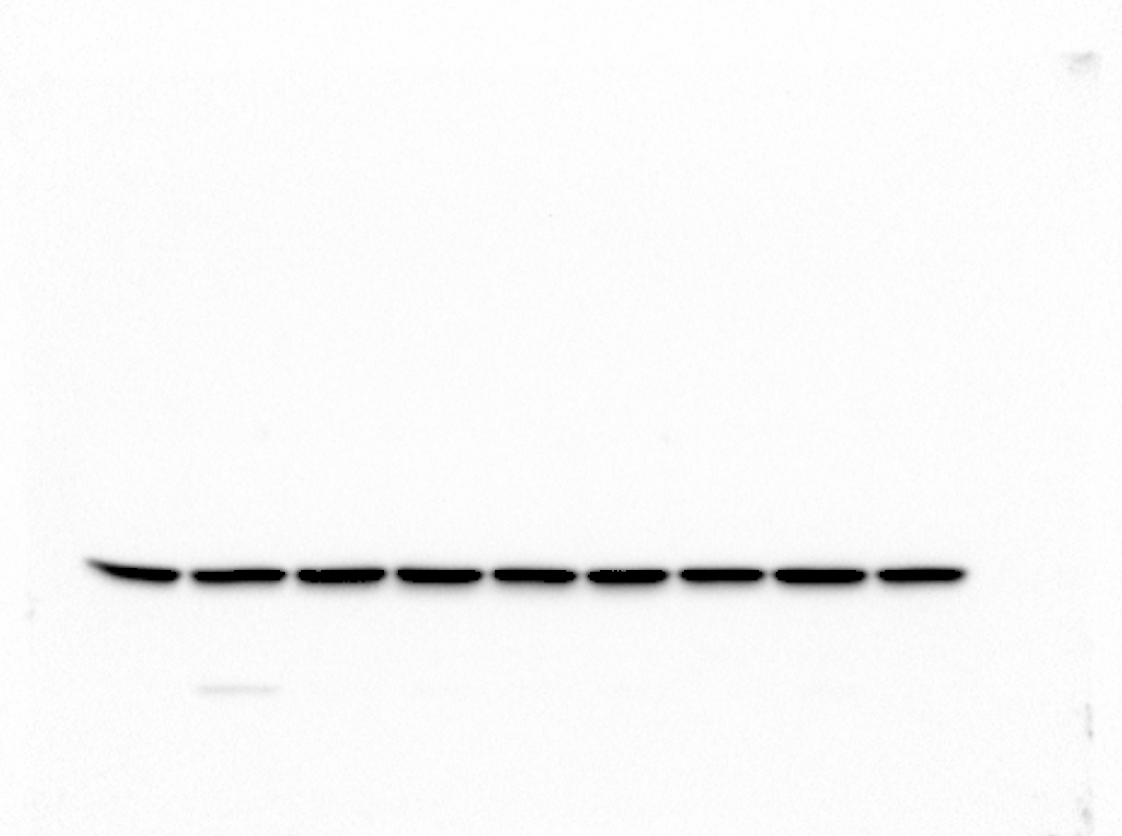

Supplement: Figure 1—source data 1. [file elife-68487-fig1-data1.zip › Figure 1-source data1/U1+PMA_GAPDH.tif]

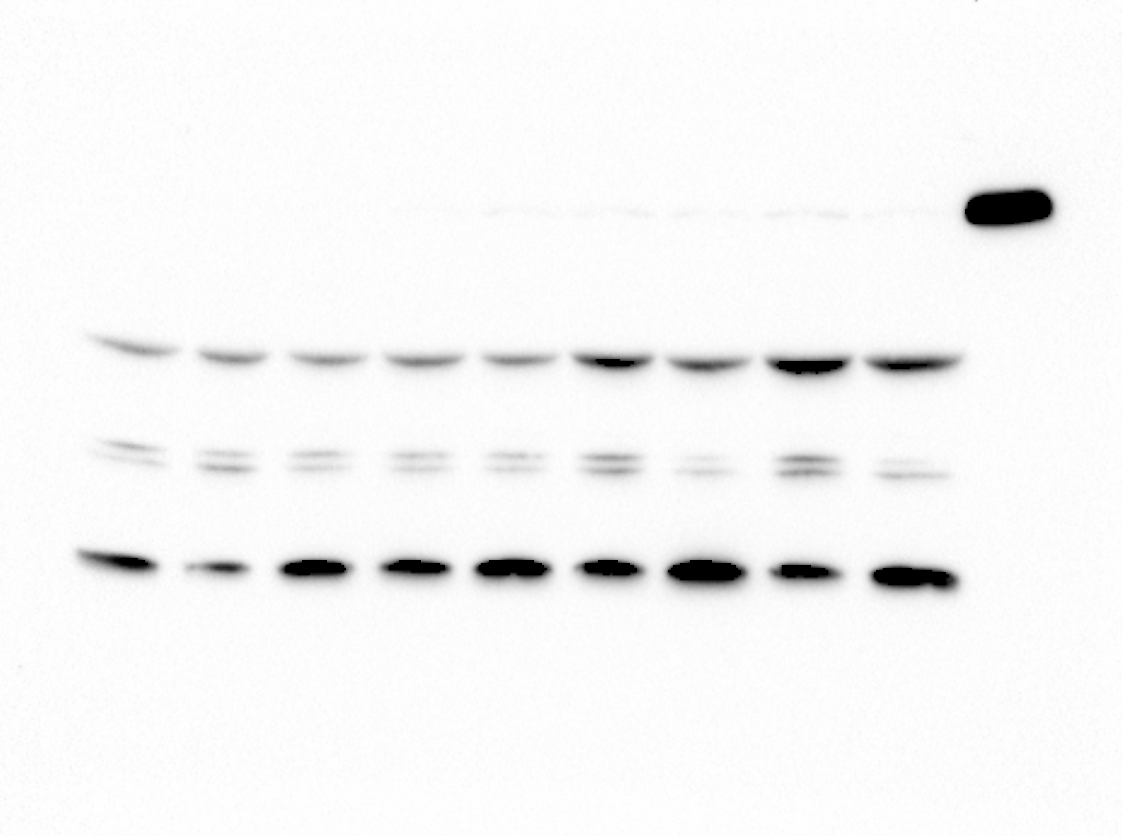

Supplement: Figure 1—source data 1. [file elife-68487-fig1-data1.zip › Figure 1-source data1/U1+PMA_CTH.tif]

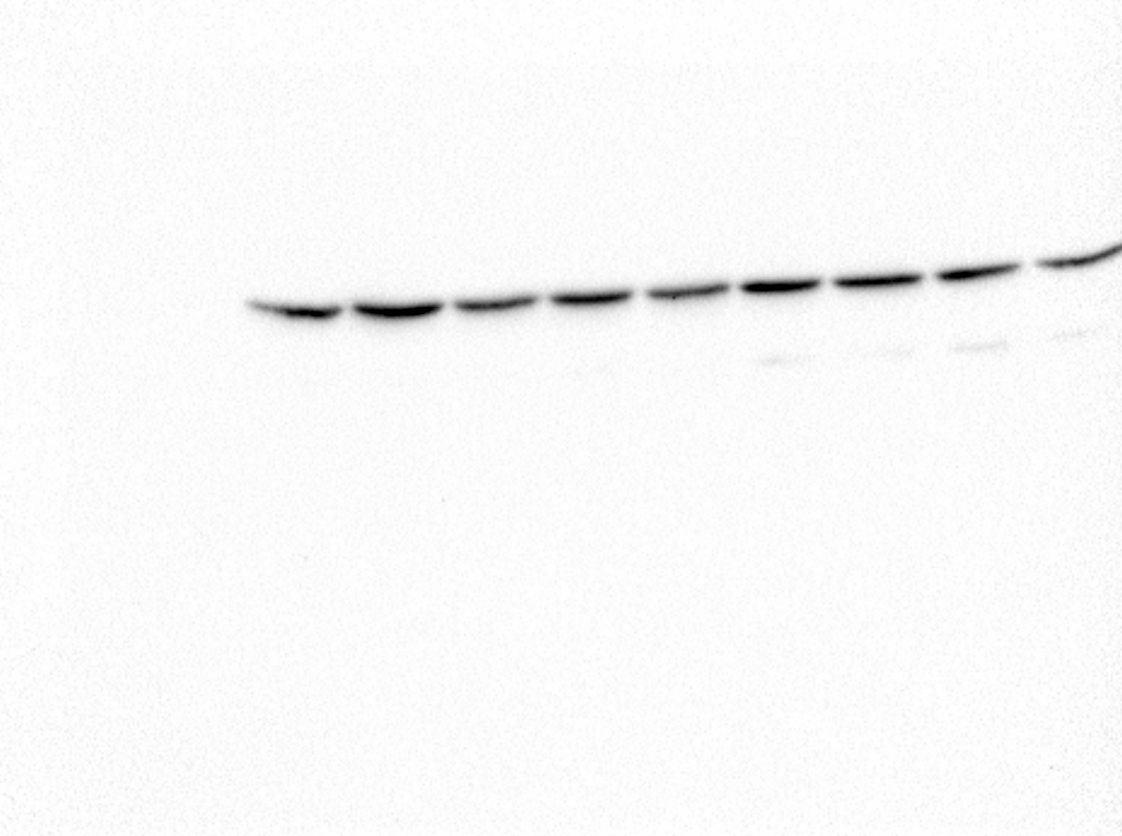

Supplement: Figure 1—source data 1. [file elife-68487-fig1-data1.zip › Figure 1-source data1/U1+PMA_CBS.tif]

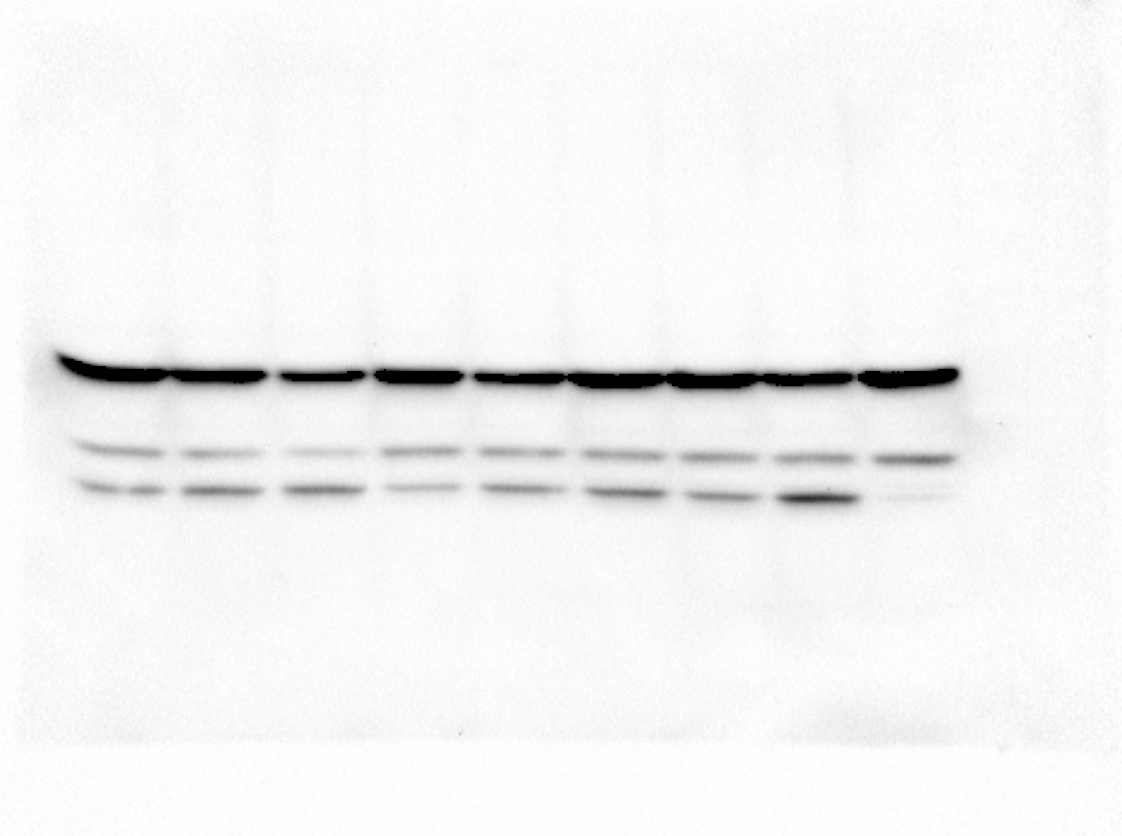

Supplement: Figure 1—source data 2. [file elife-68487-fig1-data2.zip › Figure 1-source data 2/U937+PMA_GAPDH.tif]

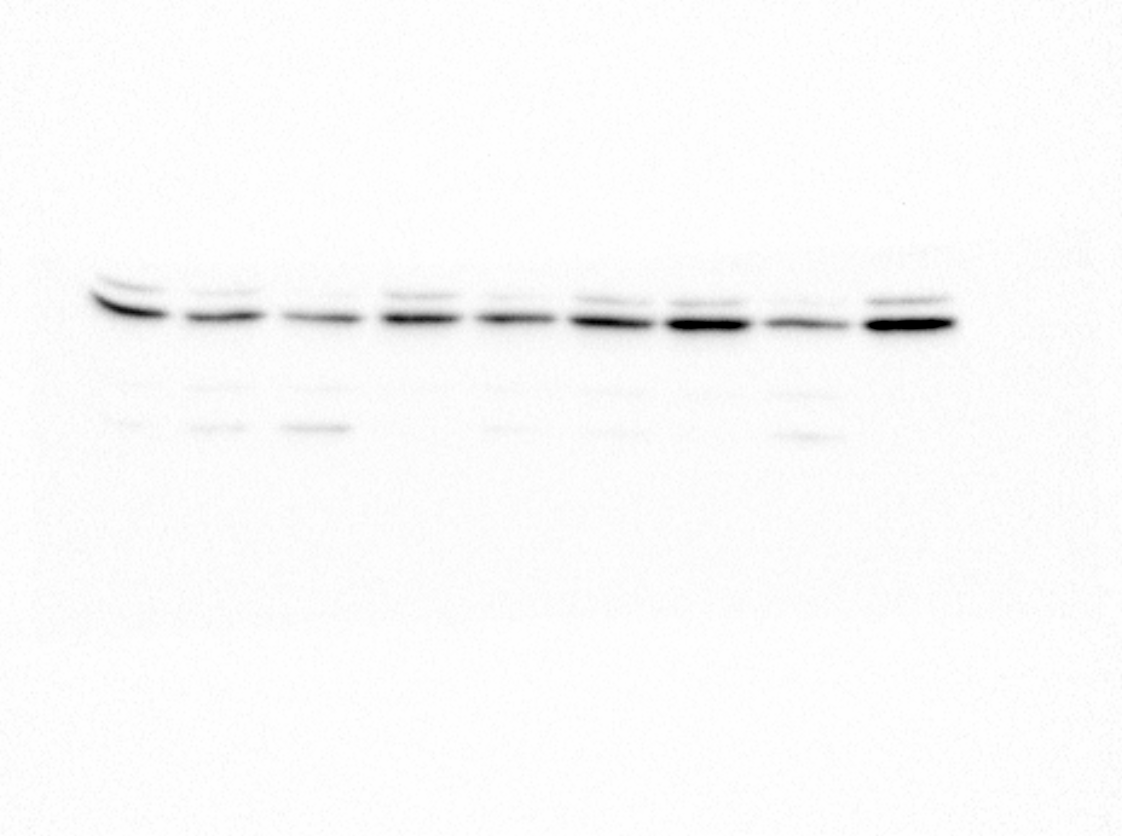

Supplement: Figure 1—source data 2. [file elife-68487-fig1-data2.zip › Figure 1-source data 2/U937+PMA_MPST.tif]

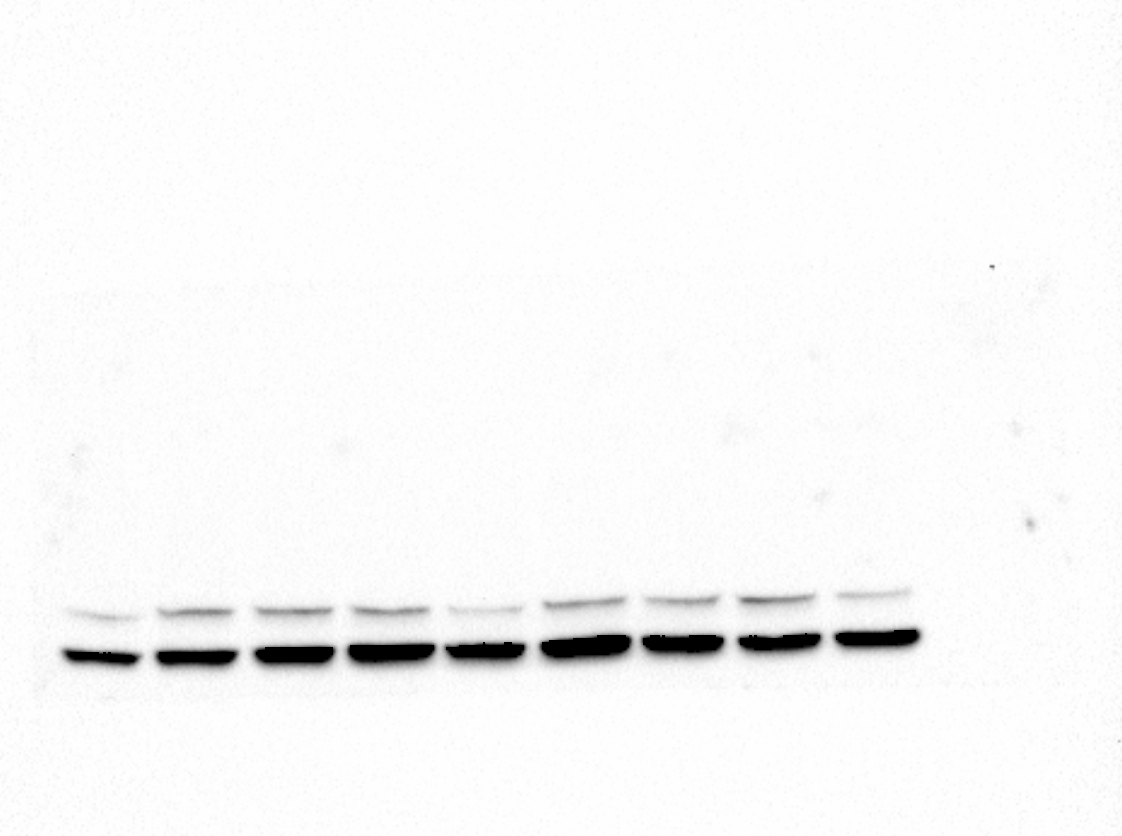

Supplement: Figure 1—source data 2. [file elife-68487-fig1-data2.zip › Figure 1-source data 2/U937+PMA_CBS.tif]

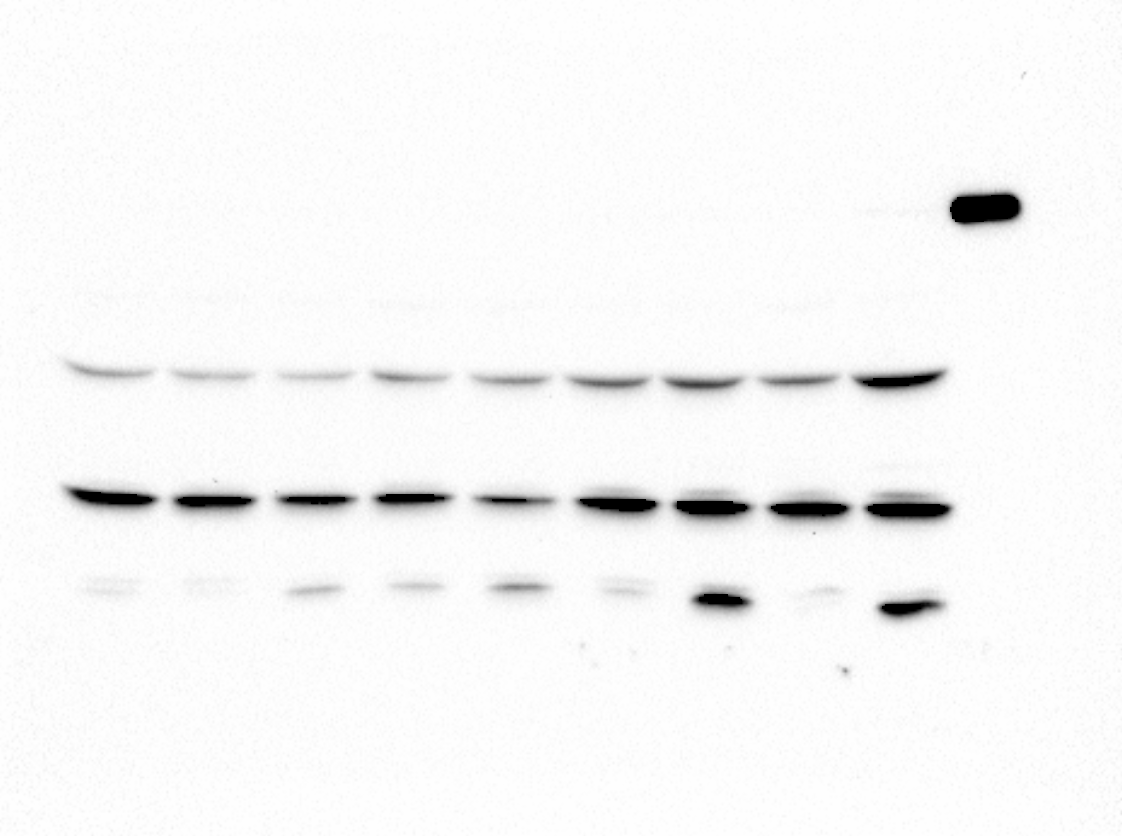

Supplement: Figure 1—source data 2. [file elife-68487-fig1-data2.zip › Figure 1-source data 2/U937+PMA_CTH.tif]

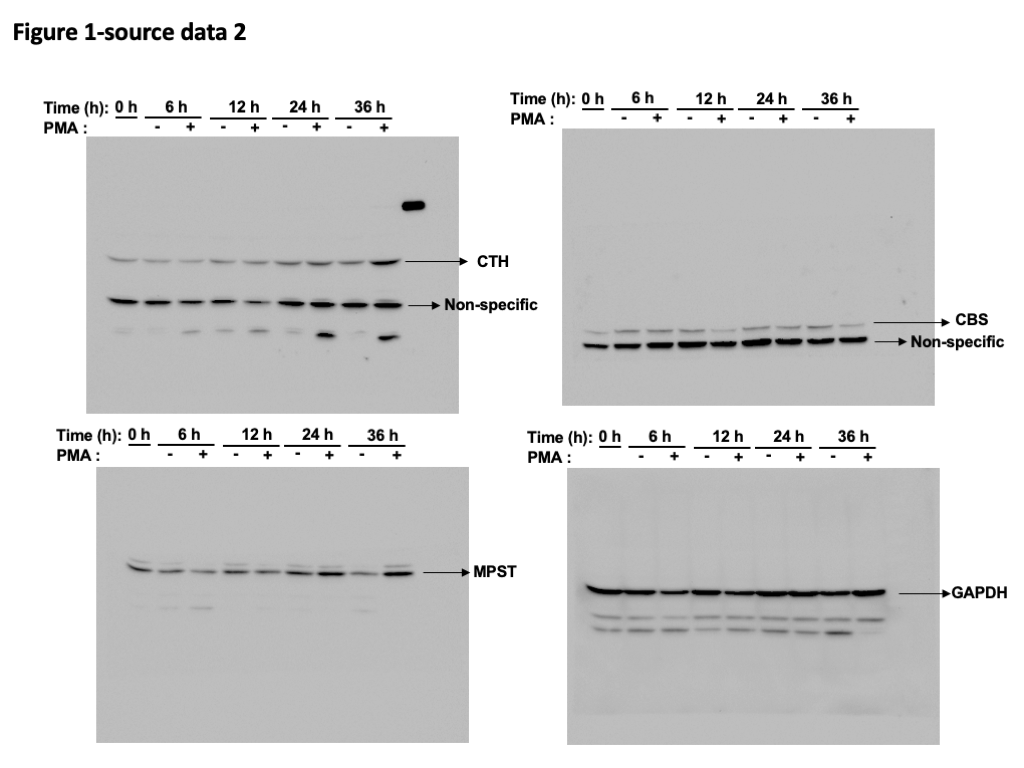

Supplement: Figure 1—source data 2. [file elife-68487-fig1-data2.zip › Figure 1-source data 2/Figure 1-source data 2.tiff]

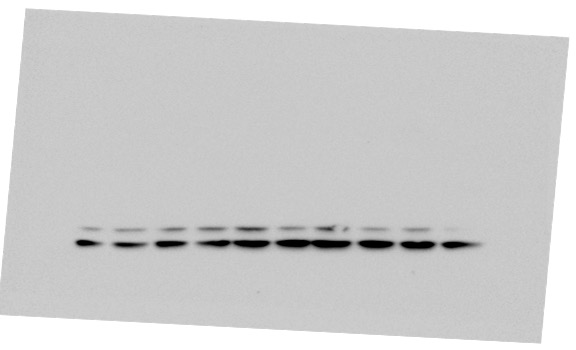

Supplement: Figure 1—figure supplement 1—source data 1. [file elife-68487-fig1-figsupp1-data1.zip › Figure 1-figure supplement 1-source data 1/J1.1+PMA_MPST.jpg]

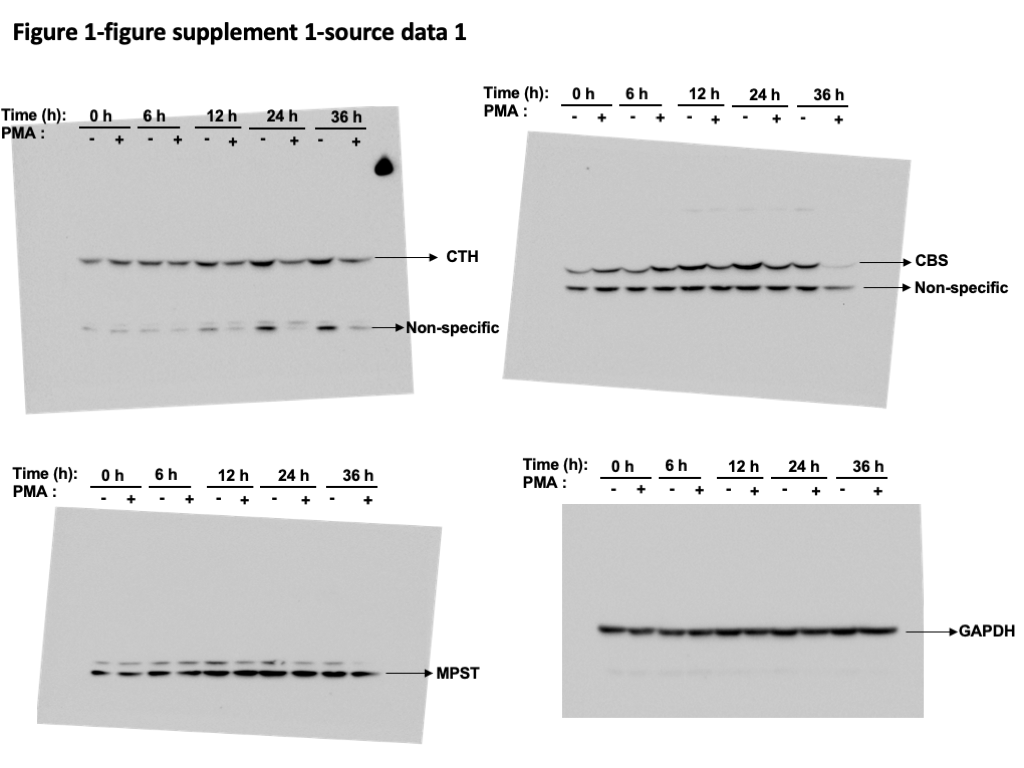

Supplement: Figure 1—figure supplement 1—source data 1. [file elife-68487-fig1-figsupp1-data1.zip › Figure 1-figure supplement 1-source data 1/Figure 1-figure supplement 1-source data 1.tiff]

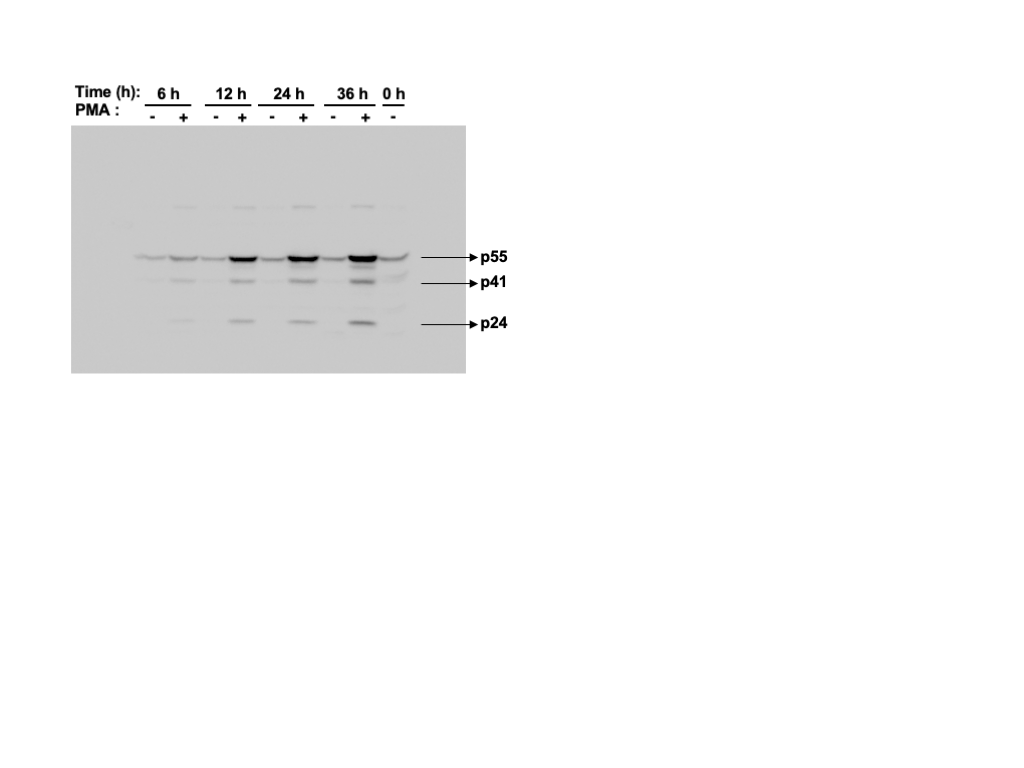

Supplement: Figure 1—figure supplement 1—source data 1. [file elife-68487-fig1-figsupp1-data1.zip › Figure 1-figure supplement 1-source data 1/Figure 1-figure supplement 1-source data 1_p24.tiff]

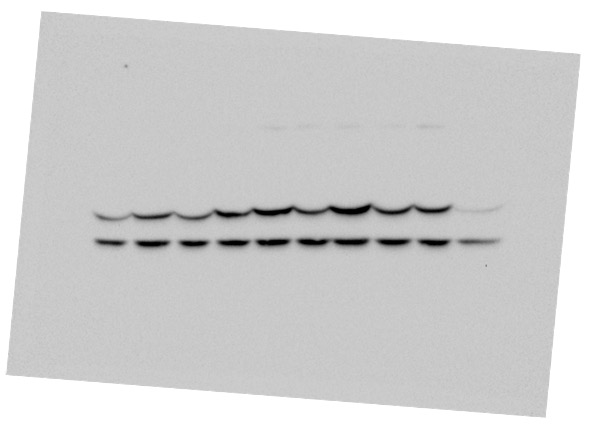

Supplement: Figure 1—figure supplement 1—source data 1. [file elife-68487-fig1-figsupp1-data1.zip › Figure 1-figure supplement 1-source data 1/J1.1+PMA_CBS.jpg]

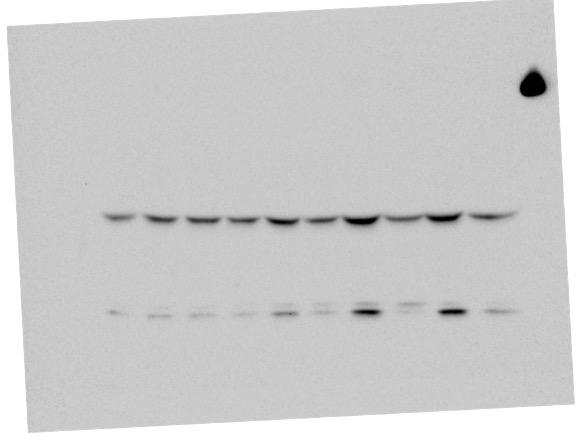

Supplement: Figure 1—figure supplement 1—source data 1. [file elife-68487-fig1-figsupp1-data1.zip › Figure 1-figure supplement 1-source data 1/J1.1+PMA_CTH.jpg]

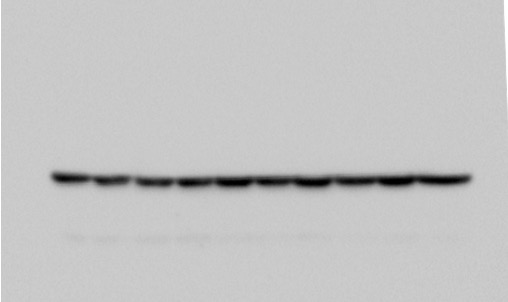

Supplement: Figure 1—figure supplement 1—source data 1. [file elife-68487-fig1-figsupp1-data1.zip › Figure 1-figure supplement 1-source data 1/J1.1+PMA_GAPDH.jpg]

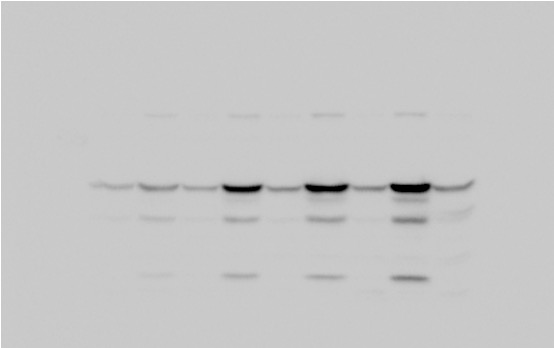

Supplement: Figure 1—figure supplement 1—source data 1. [file elife-68487-fig1-figsupp1-data1.zip › Figure 1-figure supplement 1-source data 1/J1.1+PMA_p24.jpg]

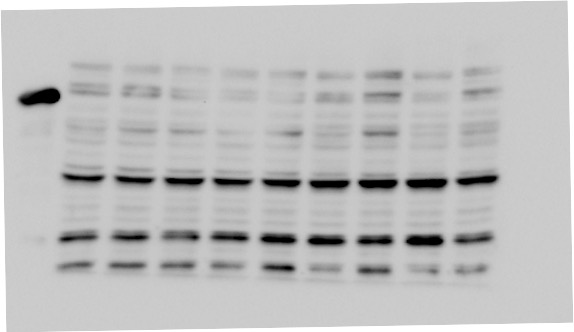

Supplement: Figure 1—figure supplement 1—source data 2. [file elife-68487-fig1-figsupp1-data2.zip › Figure 1-figure supplement 1-source data 2/Jurkat_CTH.jpg]

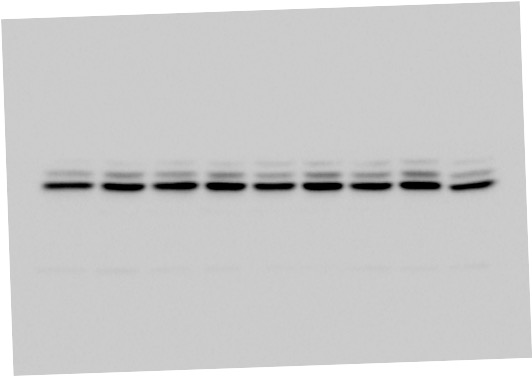

Supplement: Figure 1—figure supplement 1—source data 2. [file elife-68487-fig1-figsupp1-data2.zip › Figure 1-figure supplement 1-source data 2/Jurkat+PMA_MPST.jpg]

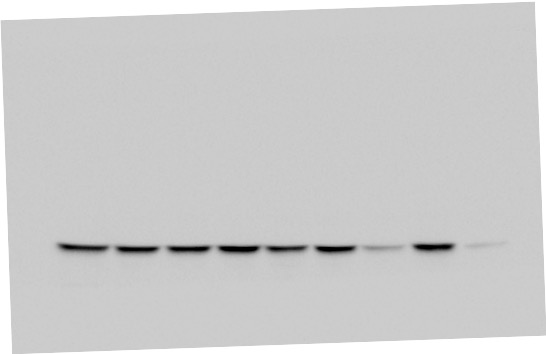

Supplement: Figure 1—figure supplement 1—source data 2. [file elife-68487-fig1-figsupp1-data2.zip › Figure 1-figure supplement 1-source data 2/Jurkat+PMA_CBS.jpg]

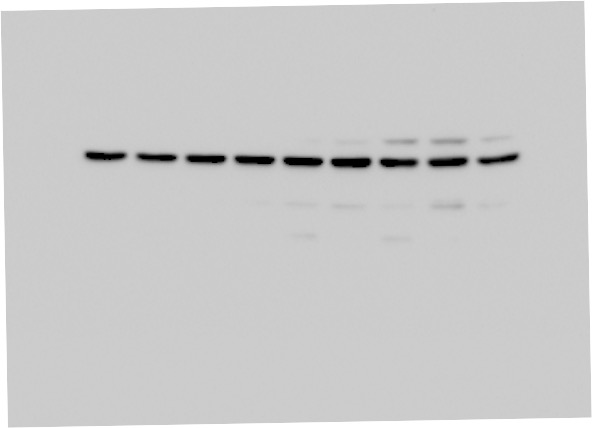

Supplement: Figure 1—figure supplement 1—source data 2. [file elife-68487-fig1-figsupp1-data2.zip › Figure 1-figure supplement 1-source data 2/Jurkat+PMA_GAPDH.jpg]

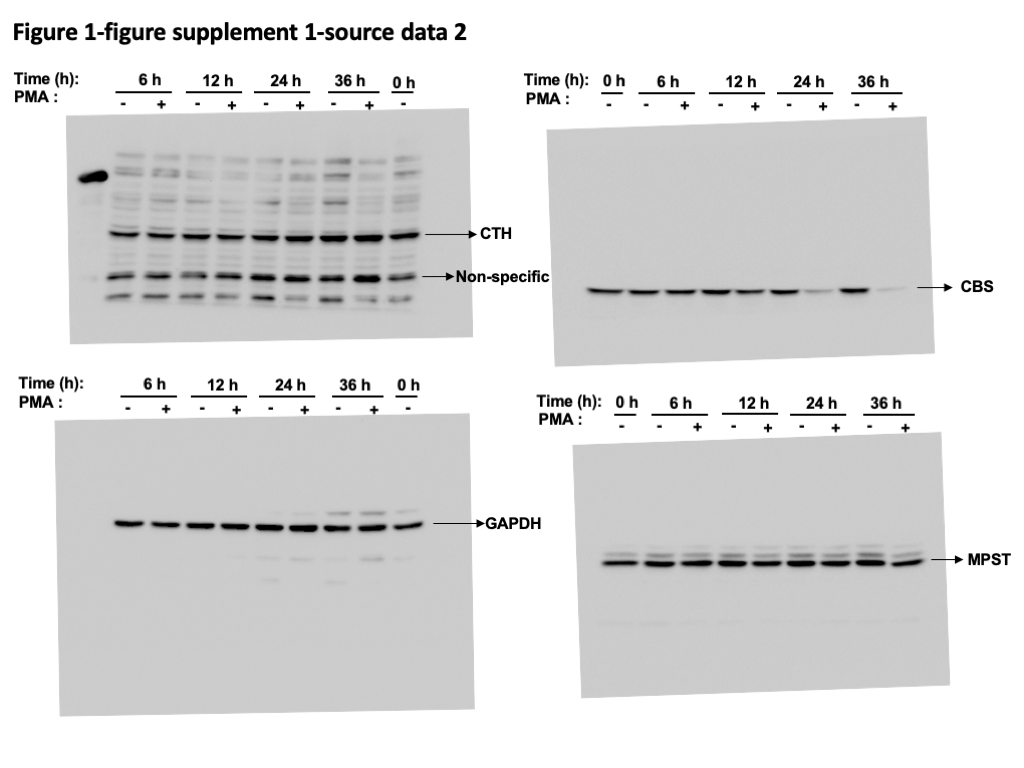

Supplement: Figure 1—figure supplement 1—source data 2. [file elife-68487-fig1-figsupp1-data2.zip › Figure 1-figure supplement 1-source data 2/Figure 1-figure supplement 1-source data 2.tiff]

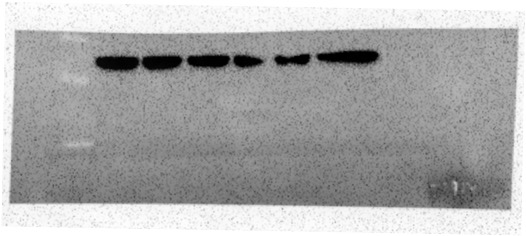

Supplement: Figure 2—source data 1. [file elife-68487-fig2-data1.zip › Figure 2-source data 1/U1-shCTH_GAPDH.jpg]

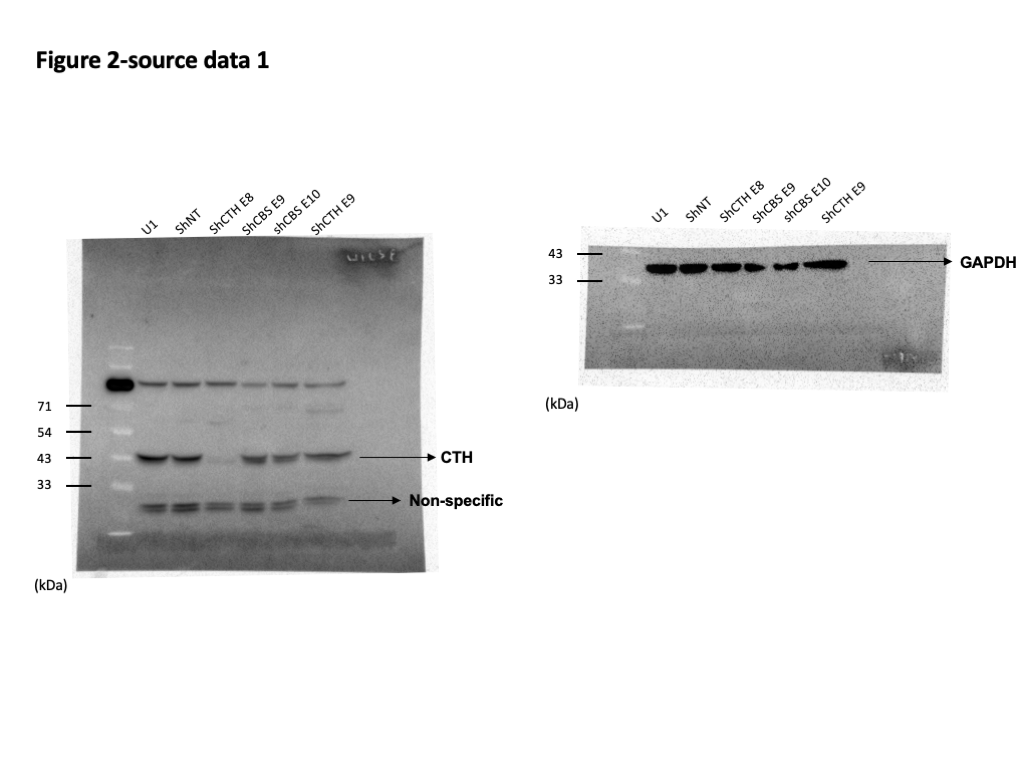

Supplement: Figure 2—source data 1. [file elife-68487-fig2-data1.zip › Figure 2-source data 1/Figure 2-source data 1.tiff]

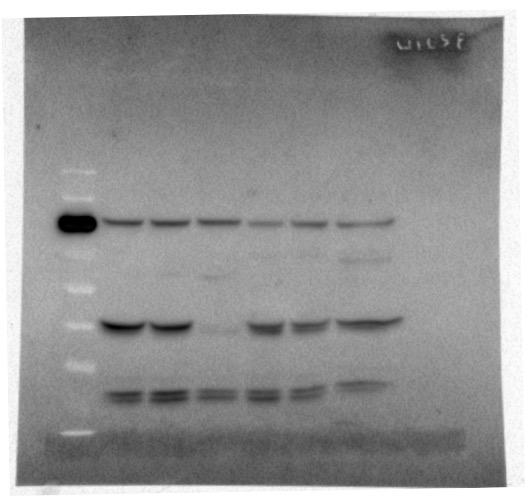

Supplement: Figure 2—source data 1. [file elife-68487-fig2-data1.zip › Figure 2-source data 1/U1-shCTH_CTH.jpg]

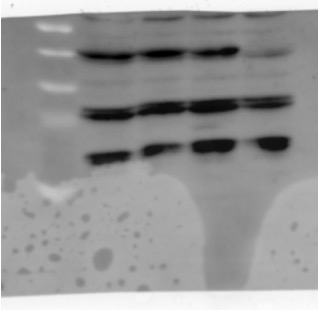

Supplement: Figure 2—figure supplement 1—source data 1. [file elife-68487-fig2-figsupp1-data1.zip › Figure 2-figure supplement 1-source data 1/J1.1_CTH.jpg]

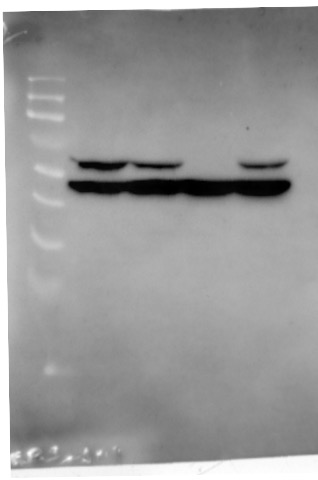

Supplement: Figure 2—figure supplement 1—source data 1. [file elife-68487-fig2-figsupp1-data1.zip › Figure 2-figure supplement 1-source data 1/J1.1_CBS.jpg]

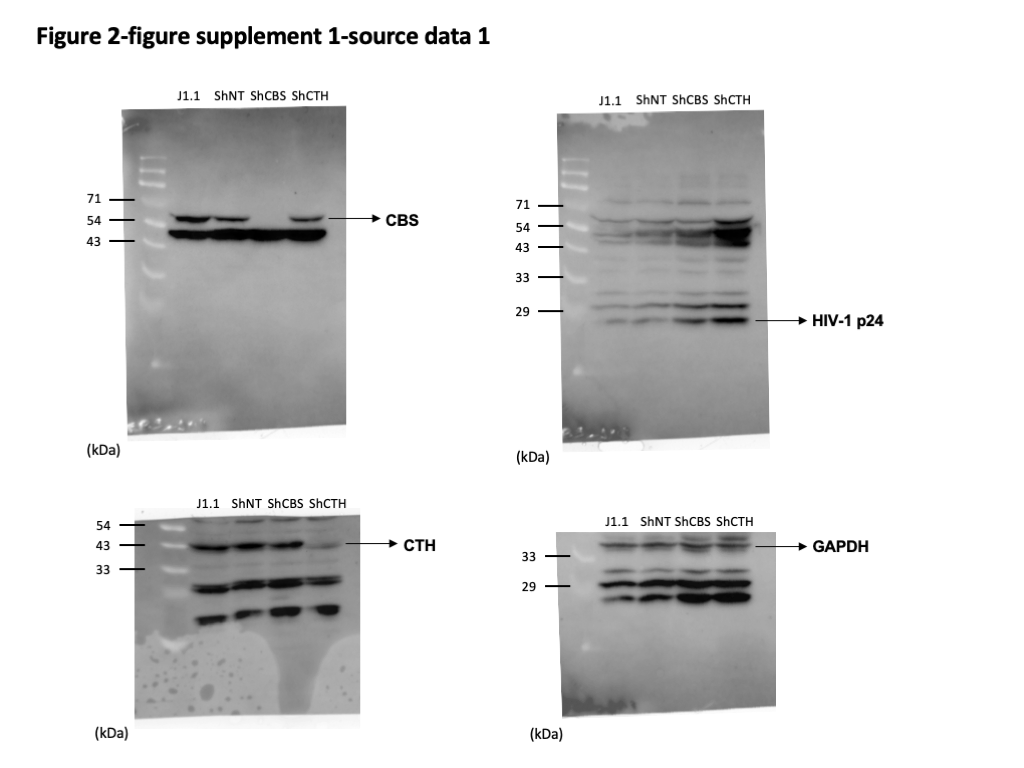

Supplement: Figure 2—figure supplement 1—source data 1. [file elife-68487-fig2-figsupp1-data1.zip › Figure 2-figure supplement 1-source data 1/Figure 2-figure supplement 1-source data 1.tiff]

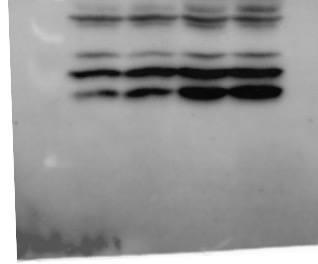

Supplement: Figure 2—figure supplement 1—source data 1. [file elife-68487-fig2-figsupp1-data1.zip › Figure 2-figure supplement 1-source data 1/J1.1_GAPDH.jpg]

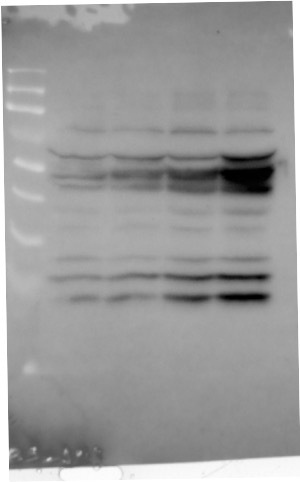

Supplement: Figure 2—figure supplement 1—source data 1. [file elife-68487-fig2-figsupp1-data1.zip › Figure 2-figure supplement 1-source data 1/J1.1_p24.jpg]

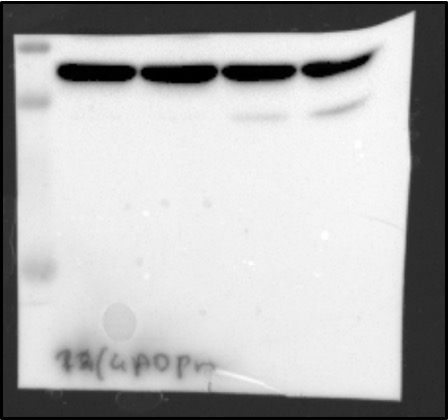

Supplement: Figure 5—source data 2. [file elife-68487-fig5-data2.zip › Figure 5-source data 2/U1+GYY+PMA_GAPDH.jpg]

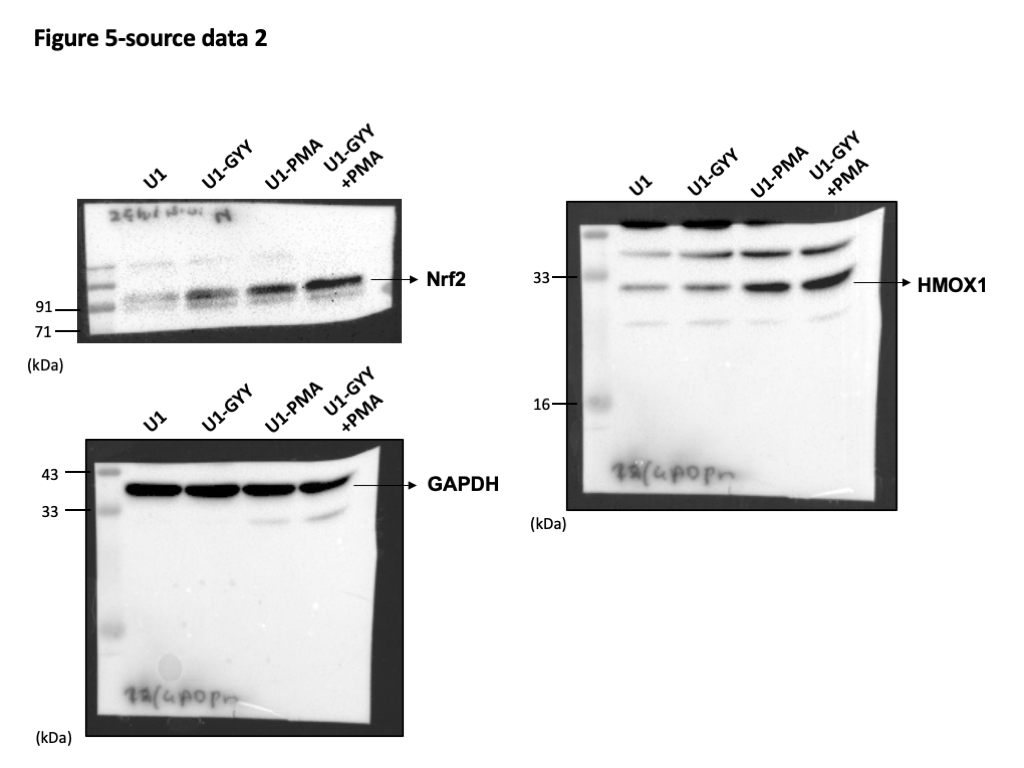

Supplement: Figure 5—source data 2. [file elife-68487-fig5-data2.zip › Figure 5-source data 2/Figure 5-source data 2.tiff]

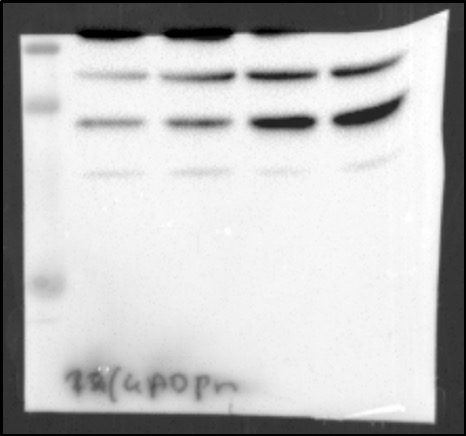

Supplement: Figure 5—source data 2. [file elife-68487-fig5-data2.zip › Figure 5-source data 2/U1+GYY+PMA_HMOX1.jpg]

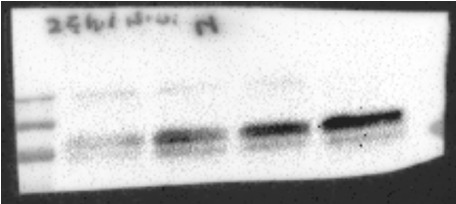

Supplement: Figure 5—source data 2. [file elife-68487-fig5-data2.zip › Figure 5-source data 2/U1+GYY+PMA_Nrf2.jpg]

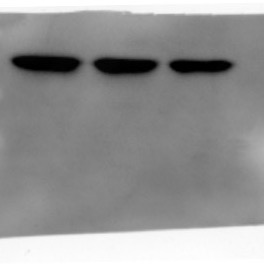

Supplement: Figure 5—source data 3. [file elife-68487-fig5-data3.zip › Figure 5-source data 3/U1+GYY+PMA_GAPDH.jpg]

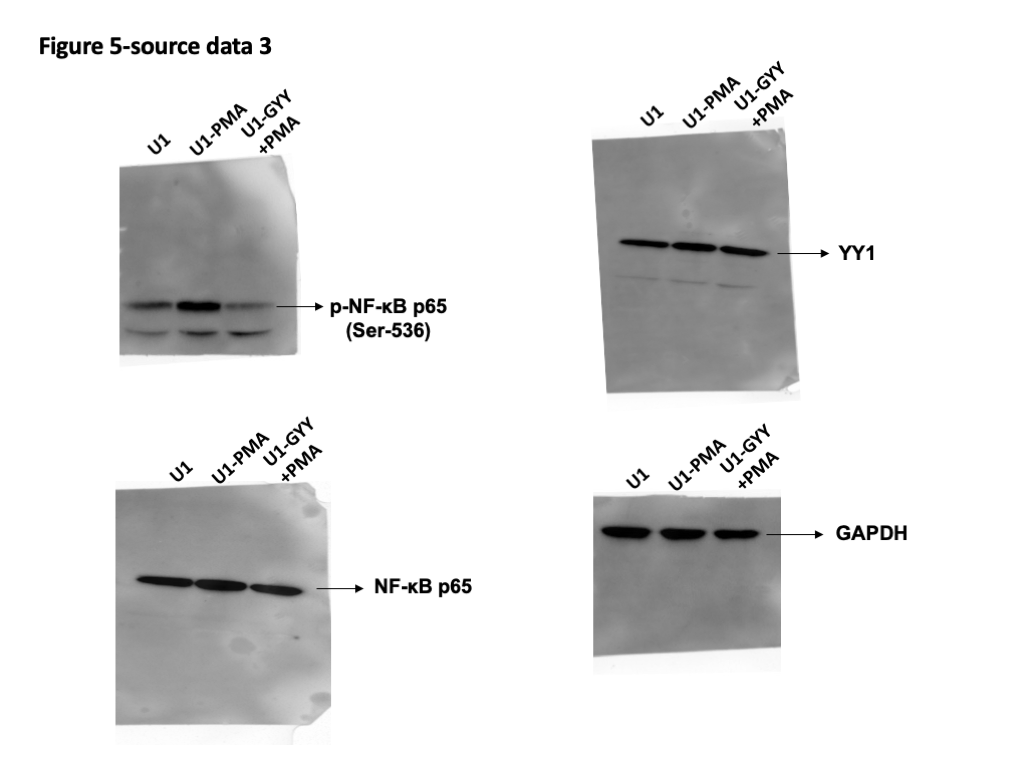

Supplement: Figure 5—source data 3. [file elife-68487-fig5-data3.zip › Figure 5-source data 3/Figure 5-source data 3.tiff]

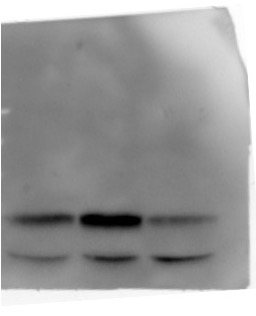

Supplement: Figure 5—source data 3. [file elife-68487-fig5-data3.zip › Figure 5-source data 3/U1+GYY+PMA_p-p65.jpg]

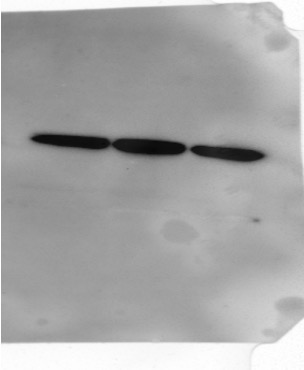

Supplement: Figure 5—source data 3. [file elife-68487-fig5-data3.zip › Figure 5-source data 3/U1+GYY+PMA_p65.jpg]

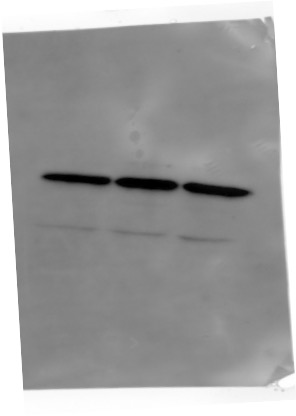

Supplement: Figure 5—source data 3. [file elife-68487-fig5-data3.zip › Figure 5-source data 3/U1+GYY+PMA_YY1.jpg]
